# Population pharmacokinetics–pharmacodynamics of sunitinib in pediatric patients with solid tumors

**DOI:** 10.1007/s00280-020-04106-z

**Published:** 2020-07-04

**Authors:** Erjian Wang, Steven G. DuBois, Cynthia Wetmore, Reza Khosravan

**Affiliations:** 1 Pfizer Global Product Development, 10646 Science Center Drive, CB10, La Jolla, CA 92121 USA; 2 Department of Pediatrics, Harvard Medical School and Dana-Farber/Boston Children’s Cancer and Blood Disorders Center, Boston, MA USA; 3 Center for Cancer and Blood Disorders, Phoenix Children’s Hospital, and Department of Child Health, University of Arizona College of Medicine-Phoenix, Phoenix, AZ USA

**Keywords:** Children, Pharmacokinetic, Pharmacodynamic, Safety, Solid tumor, Sunitinib

## Abstract

**Purpose:**

The safety profile of sunitinib in children, including the impact of sunitinib exposure on safety endpoints, was assessed using population pharmacokinetic (PK) and pharmacokinetic–pharmacodynamic (PK–PD) models.

**Methods:**

Data were from two clinical studies in 59 children with solid tumors (age range 2–21 years, 28 male/31 female, body weight range 16.2–100 kg, body surface are [BSA] range 0.7–2.1 m^2^). Analysis of covariates that affected PK and PD parameters was conducted using a nonlinear mixed-effects model. Safety and tolerability endpoints were absolute neutrophil count, hepatic transaminases, diastolic blood pressure, hemoglobin, lymphocyte count, platelet count, white blood cell count, hand-foot syndrome, fatigue, nausea, intracranial hemorrhage, and vomiting.

**Results:**

The models well described the time courses of concentrations of sunitinib and its primary active metabolite SU012662, as well as safety and tolerability endpoints. In PK models for sunitinib and SU012662, BSA was the only covariate that statistically significantly affected apparent clearance (CL/F) and apparent central volume of distribution (Vc/F). Higher BSA was associated with greater CL/F and Vc/F. No statistically significant covariates were identified in the PK–PD models. For safety endpoints that had a sufficient number of adverse events, a higher probability of adverse events was associated with higher average plasma sunitinib concentrations.

**Conclusion:**

In PK models, BSA was the only covariate that affected major PK parameters of sunitinib and SU012662. Based on analysis of safety and tolerability endpoints, the PK–PD relationships were mainly driven by sunitinib plasma exposures and were not affected by age, sex, respective baseline safety endpoint values, baseline Eastern Cooperative Oncology Group performance status, or body size.

**Trial registration:**

ClinicalTrials.gov: NCT00387920 (registered October 13, 2006), NCT01462695 (registered October 31, 2011).

**Electronic supplementary material:**

The online version of this article (10.1007/s00280-020-04106-z) contains supplementary material, which is available to authorized users.

## Introduction

Sunitinib is a multi-target tyrosine kinase inhibitor approved worldwide for the treatment of a range of advanced adult cancers [1–6]. Sunitinib has a well-established safety profile in adults, with the most common adverse events (AEs) including fatigue/asthenia, diarrhea, mucositis/stomatitis, nausea, decreased appetite/anorexia, hypertension, vomiting, abdominal pain, hand-foot syndrome, bleeding events, dysgeusia/altered taste, dyspepsia, and thrombocytopenia [5, 6]. Rarer events with sunitinib treatment include left ventricular fraction (LVEF) decrease (incidence of 7% in patients with renal cell carcinoma), dose-dependent prolonged QT interval which may lead to an increased risk of ventricular arrhythmias such as Torsade de Pointes in < 0.1% of patients, and seizures in < 1% of patients [5]. To date, however, there is limited experience of sunitinib in pediatric patients, and the safety profile has not been fully defined [7–12].

Population pharmacokinetic (PK) models have been previously developed in healthy adults or adults with solid tumors to assess the PK of sunitinib and its active metabolite SU012662 that examined covariates that might explain the variability in exposure of sunitinib and SU012662 and to make predictions on their efficacy and safety [13–16]. Based on regulatory proposals that promote the use of model-informed drug development in pediatric patients [17, 18], population PK and pharmacokinetic–pharmacodynamic (PK–PD) models in these patients may also help to explore further the safety profile of sunitinib and, in particular, the impact of sunitinib exposure on safety endpoints. The objectives of the current study were to develop a population PK model for sunitinib and SU012662, identify covariates that account for the inter-individual variability in the PK of sunitinib and SU012662, and develop sequential PK–PD and/or exposure–response models with respect to key safety and tolerability endpoints using sunitinib PK model post hoc predictions.

## Materials and methods

### Patient population

Data were pooled from two Phase I/II clinical trials in pediatric patients with solid tumors (studies ADVL0612 and ACNS1021), including predominantly high-grade glioma, ependymoma, brain stem glioma, or sarcoma. Details of these trials have been published previously [8–10]. The original trials were approved by the institutional review boards at all participating centers and The National Cancer Institute Pediatric Central institutional review board also approved study ACNS1021. All participants in the original trials or their parent/legal guardian signed a document of informed consent and assent was obtained as appropriate. A detailed description of the eligibility and exclusion criteria can be found in the Online Resource Methods. Patients were between 18 months and 22 years of age and received sunitinib at a starting dose of 15 or 20 mg/m^2^ on a schedule of 4 weeks on treatment followed by 2 weeks off treatment (schedule 4/2) [8–10]. In total, data for analysis were available from 59 patients (ADVL0612, *n* = 35; ACNS1021, *n* = 24).

### Bioanalytical methods

Sample collections (1.5 mL) for PK analysis were completed at pre-specified visits. For study ADVL0612, the series of samples for PK analysis was conducted at 0, 1, 2, 4, 6, 8–10, and 24–48 h post-dose on day 1 of cycle one and pre-dose (trough) assessments were made on days 7, 14, 21, and 28 of cycle one. For study ACNS1021, the series of samples for PK analysis were taken at 2, 4, 6–8, and 24 ± 1 h post-dose on day 1 of cycle one. In addition, pre-dose (trough) assessments were made on days 7, 14, 21, and 28 of cycle one, and days 1 and 28 of cycle two. The majority of the safety assessment data that were to be used for PK–PD modeling were captured at each study visit, but some safety assessments were performed less frequently per the specific requirements of each study protocol. Plasma samples were analyzed for the determination of sunitinib and SU012662 concentrations using a sensitive, specific, and validated liquid chromatography with tandem mass spectrometry assay (BASi, West Lafayette, IN), as previously published [8–10]. For study ADVL0612, calibration standard responses met acceptance criteria over the range of 1–200 ng/mL for sunitinib and 1–100 ng/mL for SU012662, using a quadratic weighted (l/concentration^2^) regression. The lower limit of quantification (LLOQ) for both sunitinib and SU012662 was 1 ng/mL. The between-day assay accuracy, expressed as percent relative error, for quality control (QC) concentrations, ranged from − 3.3–1.3% for sunitinib and − 1–3.8% for SU012662 for the low, medium, high, and diluted QC samples. Assay precision, expressed as the between-day percent coefficient of variation of the mean estimated concentrations of QC samples, was ≤ 5.1% for the low (3.00 ng/mL), medium (100 ng/mL), and high (150 ng/mL) concentrations of sunitinib, and was ≤ 9.5% for the low (3.00 ng/mL), medium (50.0 ng/mL), high (75.0 ng/mL), and diluted (15.0 ng/mL) concentrations of SU012662. For study ACNS1021, calibration standard responses met acceptance criteria over the range of 0.100–60.0 ng/mL for sunitinib, and 0.100–20.0 ng/mL for SU012662. The LLOQ for both sunitinib and SU012662 was 0.100 ng/mL. The between-day assay accuracy for QC concentrations ranged from 3.7 to 7.3% for sunitinib and − 3.3%–5.3% for SU012662 for low, medium, high, and diluted QC samples. Assay precision was ≤ 7.5% for low (0.300 ng/mL), medium (30.0 ng/mL), high (45.0 ng/mL), and diluted (9.00 ng/mL after dilution) concentrations of sunitinib, and was ≤ 13.7% for the low (0.300 ng/mL), medium (9.00 ng/mL), high (15.0 ng/mL), and diluted (3.00 ng/mL after dilution) concentrations of SU012662.

### Model development

The PK and PK–PD modeling approaches were described in detail in the analysis plan before initiating the analyses. Regulatory guidance and quality control were taken into account. A systematic multistep approach to model development consisted of base model development, random effects model development, full model development, final model development, assessment of model adequacy (goodness-of-fit), and assessment of model predictive performance (validation). Analysis was conducted using nonlinear mixed-effects modeling methodology, as implemented in NONMEM (v7.1.2, University of California at San Francisco, CA), and using the first-order conditional estimation method with interaction to estimate all parameters. During model development, the goodness-of-fit of different models to the data were evaluated using change in the objective function, visual inspection of different scatterplots, precision of the parameter estimates, and decreases in inter-individual and residual variability. The base model consisted of a two-compartment model with first-order absorption and lag time (*t*_lag_) to fit sunitinib and SU012662 concentrations. The type of base models used for the PK–PD modeling portion were transit compartments in series with feedback loop models or indirect response models, as used previously [16]. Sequential PK–PD models for the safety and tolerability endpoints were built using the final PK model–predicted sunitinib concentrations. SU012662 data were not included in this process because a previous study showed that inclusion of the predicted SU012662 concentrations did not improve the model objective function value (OFV) [16]. During the modeling portion, safety endpoints were used when there was a sufficient number of patients with PK and PD data (i.e., at least two patients with both PK and PD data, either both baseline and post-baseline for continuous safety endpoints, or post-baseline for categorical safety endpoints). The safety and tolerability endpoints assessed were related to the most common safety events observed with sunitinib use [5, 6], which were absolute neutrophil count (ANC), alanine transaminase (ALT), aspartate transaminase (AST), diastolic blood pressure (BP), hemoglobin, lymphocyte count, platelet count, white blood cell (WBC) count, hand-foot syndrome, fatigue, nausea, intracranial hemorrhage, and vomiting. For categorical safety endpoints, PK–PD modeling was not used. Instead, the relationships between the average daily plasma exposures (i.e., the accumulated dose divided by the time after the first dose, divided by the individual sunitinib clearance × 1000) up to the time of earliest worst grade Common Terminology Criteria for Adverse Events (CTCAE) and the incidence rate were explored by looking at different CTCAE grade incidence rates at the lower and upper halves for the mean plasma exposures, as well as using ordered logistic regression approaches, where there was a sufficient number of events (i.e., more than one event). Interindividual variability in the PK or PK–PD parameters was modeled using multiplicative exponential random effects of the form *θ*_*i*_ = *θ* × *e*^*ηi*^, where θ is the typical or central value of the parameter, and the empirical Bayes prediction of the interindividual random effect (*η*) was a random variable normally distributed with a mean 0 and variance *ω*^2^.

Based on prior experience, a group of potential covariates was examined with respect to the PK and PK–PD parameters (Table 1). Covariates were tested for significance in a stepwise manner using a stepwise covariate model (SCM) building procedure and statistical criteria of *α* = 0.01 for the forward inclusion step. The full model was then subjected to a backward elimination step with a statistical criterion of *α* = 0.001. In the SCM approach, linear and power functions were tested for any continuous covariates on PK parameters, with the SCM model reporting the best one selected (i.e., the one with the lower OFV). Baseline body weight and body surface area (BSA) were examined to ensure that the body size measure with the largest effect was included in the final model, and other measures related to body size that were highly correlated with body weight or BSA, such as lean body weight, body mass index, and height, were not included in the covariate analysis. BSA was calculated based on the method of DuBois and DuBois [i.e., BSA = 0.20247 × height (m)^0.725^ × weight (kg)^0.425^]. No adaptation was required because no patients had undergone prior amputation. In addition, for both sunitinib and SU012662, baseline bodyweight was tested in a separate SCM run where BSA was replaced with bodyweight and the final model with the lower OFV was selected.

**Table 1:** Covariates considered in the pharmacokinetic and pharmacokinetic–pharmacodynamic analysis

Analysis	Parameters	Covariates
PK	CL/F	Baseline body weight or BSA, sex (male or female), race (Asian or non-Asian), baseline ECOG PS (0 or ≥ 0), age
PK	Vc/F	Baseline body weight, BSA, sex (male or female), age
PK	*k*_a_	Formulation (intact capsule or sprinkle capsule contents on yogurt or apple sauce)
PK–PD	EC_50_	Baseline body weight or BSA, sex (male or female), baseline ECOG PS (0 or ≥ 0), age, baseline PD value
PD–PD	*k*_PD_	Baseline body weight or BSA, sex (male or female), baseline ECOG PS (0 or ≥ 0), age, baseline PD value

*BSA* baseline surface area, *CL/F* apparent clearance, *EC*_*50*_ concentration at half maximum effect, *ECOG PS* Eastern Cooperative Oncology group performance status, *k*_*a*_ first-order absorption rate constant, *k*_*PD*_ effect first-order rate constant*, PD* pharmacodynamic, *PK* pharmacokinetic, *Vc/F* apparent central volume of distribution

### Model validation

For the validation of the base and final models, visual predictive check (VPC) techniques comprising 1000 simulations were carried out, and the median and upper and lower bounds of the 95% prediction interval (PI) for PK or PD profiles were compared against the observed data median and confidence intervals (CIs). The number of observations not within the 95% PI was to remain within 5% of the total number of observations, and the mean prediction profile was expected to follow the observed mean profile. In additon, the 90% PIs for the median and the lower and upper bounds of the 95% PIs were identified to ensure that they included the observed median and 95% CI bounds. Bootstrapping techniques (1000 boostrap datasets) were applied to generate the nominal 95% CIs around the point estimates and to confirm the 95% CIs generated by the base or final models based on asymptotic standard errors generated from the NONMEM covariance step.

## Results

The final dataset comprised 365 sunitinib and 340 SU012662 post-baseline measurable plasma observations from the 59 patients who received sunitinib [57 of 59 patients (96.6%) provided ≥ 2 samples for PK analysis]. Twenty-four of 389 (6.2%) samples were below the limit of quantification for sunitinib, and 49 of 389 (12.6%) for SU012662, and were not included in the analysis. Baseline patient characteristics overall and by age group (2–5, 6–11, 12–17, and 18–21 years) are shown in Table 2. Online Resource Fig. S1 shows the concentration–time profiles for each study using time after first dose.

**Table 2:** Patient baseline characteristics by age group

Age, year	*n*	Sex		Race^a^		ECOG PS^b^		Median body weight (range), kg	Median BSA (range), m^2^
		M	F	Asian	Non-Asian	0	> 0		
2–5	6	3	3	1	5	2	1	18.3 (16.2–28.7)	0.69 (0.66–0.98)
6–11	20	9	11	0	18	8	4	28.4 (17.1–56.3)	1.10 (0.72–1.48)
12–17	27	11	16	1	25	12	15	60.4 (37.1–100)	1.63 (1.27–2.14)
18–21	6	3	3	1	5	1	5	71.3 (62.5–74.5)	1.87 (1.62–1.92)
Total 2–21	59	28	31	3	53	23	25	50.4 (16.2–100)	1.47 (0.66–2.14)

*ECOG PS* Eastern Cooperative Oncology group performance status, *F* female, *M* male

^a^Race was unknown for three patients

^b^ECOG PS scores based on either ECOG PS or extrapolated from Karnofsky performance scale, with ECOG PS set to 0 and 1 for Karnofsky performance scale > 90 and ≤ 90, respectively. ECOG PS could not be determined for 11 patients

### Sunitinib and SU012662 base and final PK models

The sunitinib and SU012662 base models comprised a two-compartment model with first-order absorption and elimination rates, consistent with previous work. Based on previous observations [13], a conversion of 21% of sunitinib to SU012662 was assumed to bring the magnitude of the parameters for SU012662 to a more physiologically relevant level. In both the sunitinib and SU012662 base models, the diagnostic plots were satisfactory. The effects of extreme outliers on the population PK parameter estimates and on the diagnostic plots were tested in both models, and no outliers met the criteria for exclusion from the datasets [i.e., |conditional weighted residual (CWRES)|> 6]. Bootstrap results were consistent with the population parameter estimates, indicating that the base models were stable and the parameter estimates represented the final datasets adequately.

Using SCM, the effects of different covariates on apparent clearance (CL/F) and apparent central volume of distribution (Vc/F) in both models were examined. For sunitinib, the estimated typical values for CL/F and Vc/F were 24.1 L/h and 1070 L, respectively, and for SU012662 the values were estimated to be 10.9 L/h and 1030 L, respectively. For sunitinib, the effect of BSA on CL/F was statistically significant (*P* < 0.001) using a linear function: CL/F = 24.1 L/h · [1 + 0.557 (BSA–1.47)], and for SU012662, the effect of BSA on CL/F was statistically significant (*P* < 0.001) using a power function: CL/F = 10.9 L/h · (BSA/1.47)^0.843^. For sunitinib, the effect of BSA on Vc/F was also statistically significant (*P* < 0.001) using a power function: Vc/F = 1070 L · (BSA/1.47)^1.47^, and for SU012662, the effect of BSA was also statistically significant on Vc/F (*P* < 0.001) using a power function: Vc/F = 1030 L · (BSA/1.47)^1.72^. Therefore, for sunitinib and SU012662, higher BSA was associated with greater CL/F and Vc/F. Baseline bodyweight was also tested in a separate SCM run where BSA was replaced with bodyweight. However, the final model with BSA was the one with the lower OFV and hence was selected. The effects on PK parameters of the other covariates tested (Table 1) were not found to be statistically significant for sunitinib or SU012662.

**Fig. 1:**
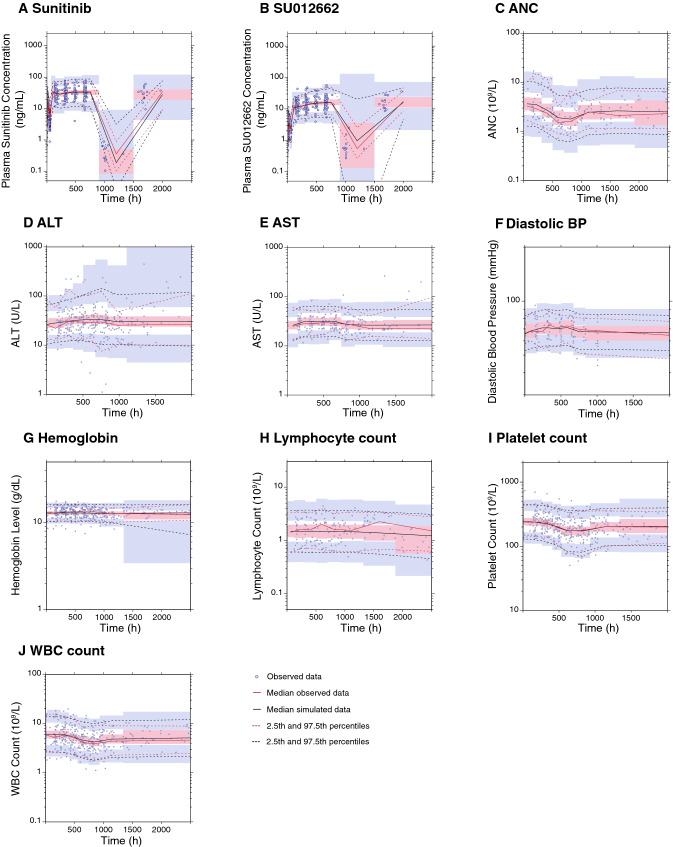
Final model prediction and variance-corrected visual predictive check plots for sunitinib and SU012662 plasma concentrations, and safety and tolerability endpoints, up to 3000 h post-dose. Visual predictive check plots for **a** sunitinib, **b** SU012662, **c** ANC, **d** ALT, **e** AST, **f** diastolic BP, **g** hemoglobin, **h** lymphocyte count, **i** platelet count, and **j** WBC count. Blue circles represent the observed data. Red lines represent the median (solid line) and 2.5th and 97.5th percentiles (dashed lines) of the observed data. Black lines represent the median (solid line) and 2.5th and 97.5th percentiles (dashed lines) of the simulated data. The 95% CIs for simulated median and each percentile are shown by pink and blue shaded areas, respectively. *ALT* alanine transaminase, *ANC* absolute neutrophil count, *AST* aspartate transaminase, *BP* blood pressure, *WBC* white blood cell

Goodness-of-fit diagnostic plots for the observed versus individual-predicted or population-predicted values, as well as CWRES versus time or population-predicted values, in the final PK models for sunitinib and SU012662 plasma concentrations demonstrated the adequacy of the models (Online Resource Fig. S2A and S2B, S3A and S3B, S4A and S4B, and S5A and S5B). Moreover, the prediction- and variance-corrected VPC plots of sunitinib and SU012662 plasma concentration demonstrated the similarity between the predicted and the observed data during the first 12 h post-dose (Online Resource Fig. S6) and up to 3000 h post-dose (Fig. 1a, b). A summary of sunitinib and SU012662 PK parameters from the final models and following bootstrapping are shown in Table 3. Bootstrap results were consistent with the population parameter estimates, indicating that the final models were stable and that the population parameter estimates represented the dataset adequately. For sunitinib, inclusion of baseline BSA as a covariate into the final model reduced interindividual variability on CL/F and Vc/F by 25.9 and 78.0%, respectively, and for SU012662, interindividual variability on CL/F and Vc/F was reduced by 28.7 and 53.9%, respectively.

**Table 3:** Sunitinib and SU012662 final model pharmacokinetic parameters summary

Parameter	Sunitinib		SU012662	
	Final model results, mean (RSE %)	Final model bootstrap^a^, median (95% CI)	Final model results, mean (RSE %)	Final model bootstrap^b^, median (95% CI)
CL/F, L/h^c^	24.1 (6.6)	23.8 (13.4–27.1)	10.9 (7.5)	11.0 (9.3–12.8)
Vc/F, L^c^	1070 (10.3)	1025 (784–1,230)	1030 (15.3)	953 (494–1231)
*k*_a_, 1/h	0.38 (31.5)	0.35 (0.21–0.57)	0.28 (36.8)	0.26 (0.13–0.38)
*t*_lag_, h	0.64 (12.8)	0.64 (0.47–0.76)	0.46 (76.9)	0.48 (0.14–0.65)
Vp/F, L	63.8 (34.3)	86.0 (45.9–776,900)	122 (72.5)	154 (26.7–445)
Q/F, L/h	0.28 (177)	0.33 (0.20–20.4)	17.8 (110)	22.9 (0.84–479)
BSA on CL/F	0.56 (19.9)	0.57 (0.31–1.04)	0.84 (30.8)	0.84 (0.41–1.27)
BSA on Vc/F	1.47 (19.6)	1.50 (1.00–1.93)	1.72 (23.5)	1.92 (1.21–3.69)
*ω*(CL/F), %	34.2 (39.1)	34.1 (27.1–61.5)	48.1 (21.9)	47.0 (34.4–60.4)
*ω*(Vc/F), %	24.1 (50.3)	20.5 (24.1–41.1)	49.9 (44.2)	51.4 (36.5–75.1)
*ω*(*k*_a_), %	87.7 (52.2)	86.5 (51.9–120)	72.4 (48.7)	65.0 (24.2–92.8)
*σ*, %	31.9 (2.7)	31.0 (24.0–39.3)	23.1 (4.7)	22.8 (19.4–27.3)

*BSA* baseline surface area, *CI* confidence interval, *CL/F* apparent clearance, *k*_*a*_ first-order absorption rate constant, *Q/F* intercompartmental clearance, *RSE* relative standard error, *t*_*lag*_ lag time, *Vc/F* apparent central volume of distribution, *Vp/F* peripheral volume of distribution, *σ* residual variability; *θ* estimate of fixed effect in NONMEM, *ω* interindividual variability

^a^For the final sunitinib model bootstrap, of 1000 replicates, 97.3% were successful

^b^For the final SU012662 model bootstrap, of 1000 replicates, 99.1% were successful

^c^Parameters are expressed for a typical patient with BSA of 1.47 m^2^

### PK–PD modeling of safety and tolerability endpoints

PK–PD models were based solely on sunitinib data, as described previously [16]. For ANC, hemoglobin, lymphocyte count, platelet count, and WBC count, a sequential transit compartments in series with feedback loop PK–PD model was used as the base model. For ANC, lymphocyte count, platelet count, and WBC count, the model included a maximum sunitinib effect (*E*_max_) on the proliferation rate constant (*k*_prol_) in the stem cell compartment, whereas for hemoglobin, the model included an effect first-order rate constant (k_PD_) effect on the k_prol_ constant in the stem cell compartment. For ALT, AST, and diastolic BP, a sequential indirect response PK–PD model was used as the base model. For ALT and AST, the models included a *k*_PD_ effect on the output/elimination rate constant (*k*_out_), and for diastolic BP, the model included a k_PD_ effect on the input rate constant (*k*_in_). Each of these models has been used previously to describe the time course of each endpoint following sunitinib dosing [16]. Following SCM analysis for each endpoint, no covariates (Table 1) were found to statistically significantly (*P* > 0.001) affect the concentration at half maximum effect (EC_50_) or k_PD_; therefore, the final model used was the base model.

Goodness-of-fit plots for the observed values versus individual-predicted or population-predicted values, as well as CRWES values versus time or predicted values, in the final PK–PD models for the continuous safety endpoints (ANC, ALT, AST, diastolic BP, hemoglobin, lymphocyte count, platelet count, and WBC count) demonstrated the adequacy of the models (Online Resource Fig. S2C–J, S3C–J, S4C–J, and S5C–J). Moreover, the prediction- and variance-corrected VPC plots of each safety endpoint demonstrated the similarity between the predicted and the observed data (Fig. 1c–j), further supporting that the PK–PD models described the time course of each of the safety and tolerability endpoints adequately. Summaries of the PK–PD parameters from each of the final models for the continuous safety endpoints are shown in Table 4. For each of these endpoints, the mean from the model bootstrap runs was consistent with those from the model run, indicating that the population parameter estimates from the models adequately represented the datasets.

**Table 4:** Results of the pharmacokinetic–pharmacodynamic final models

Parameter	Model results, mean (RSE %)	Bootstrap median (95% CI)
Absolute neutrophil count		
BASE, 10^9^/L	3.7 (14.9)	3.6 (2.8–4.8)
MTT, h	207 (12.2)	209 (163–249)
*E*_max_	0.16 (15.1)	0.16 (0.11–0.21)
EC_50_, ng/mL	1.8 (149)	1.7 (0.3–18.4)
POW	0.27 (31.6)	0.28 (0.16–0.41)
GAM	Fixed to 1 (NA)	Fixed to 1 (NA)
*ω*(BASE), %	50.8 (37.6)	48.8 (32.4–67.4)
*ω*(EC_50_), %	133 (294)	124 (1.3–237)
*σ*, %	38.8 (6.7)	38.1 (32.7–42.8)
Alanine transaminase		
BASE, UL	26.5 (6.6)	26.5 (23.3–30.8)
* k*_out_, 1/h	0.00559 (31.1)	0.00553 (0.00131–0.00195)
*k*_PD_, mL/ng	0.00443 (28.9)	0.00469 (0.00131–0.00873)
*ω*(BASE), %	44.7 (24.7)	44.0 (35.2–54.4)
*ω*(*k*_out_), %	70.7 (118)	62.7 (1.1–114)
*ω*(*k*_PD_), %	97.9 (56.4)	99.7 (63.5–186)
*σ*, %	33.9 (2.2)	33.6 (26.2–42.2)
Aspartate transaminase		
BASE, UL	26.2 (8.3)	26.1 (23.1–29.4)
*k*_out_, 1/h	1.7 (703)	1.5 (0.03–9.5)
*k*_PD_, mL/ng	0.00492 (32.7)	0.00487 (0.00341–0.0064)
*ω*(BASE), %	29.5 (30.0)	28.7 (17.8–38.0)
*ω*(*k*_out_), %	435 (529)	369 (4.5–648)
*ω*(*k*_PD_), %	2.1 (420)	1.5 (0.04–23.8)
*σ*, %	31.2 (2.7)	31.0 (21.7–40.4)
Diastolic blood pressure		
BASE, mmHg	65.9 (3.1)	65.5 (62.4–68.7)
*k*_out_, 1/h	0.0170 (122)	0.0176 (0.0341–62.8)
*k*_PD_, mL/ng	0.00225 (47.6)	0.00255 (0.00138–0.0056)
*ω*(BASE), %	9.6 (56.7)	9.4 (6.7–11.6)
*ω*(*k*_out_), %	117 (672)	120 (1.2–515)
*ω*(*k*_PD_), %	50.5 (227)	35.7 (0.5–103)
*σ*, %	9.4 (7.7)	9.2 (7.2–11.0)
Hemoglobin		
BASE, 10^9^/L	13.0 (1.8)	13.0 (12.6–13.4)
MTT, h	1,370 (13.0)	1,379 (800–3,080)
*k*_PD_, mL/ng	0.000317 (59.0)	0.000580 (0.0000471–0.00355)
POW	Fixed to 1 (NA)	Fixed to 1 (NA)
*ω*(BASE), %	12.1 (16.8)	12.0 (9.2–15.0)
*ω*(*k*_PD_), %	262 (35.8)	198 (59.4–490)
*σ*, %	5.8 (2.7)	5.7 (5.1–6.4)
Lymphocyte count		
BASE, 10^9^/L	1.5 (11.8)	1.5 (1.2–1.8)
MTT, h	1,990 (15.5)	1,997 (1239–3,111)
*E*_max_	1 (Fixed)	1 (Fixed)
EC_50_, ng/mL	165 (181)	153 (1.7–6.2^8^)
POW,	Fixed to 1 (NA)	Fixed to 1 (NA)
GAM	Fixed to 1 (NA)	Fixed to 1 (NA)
*ω*(BASE), %	49.3 (54.3)	48.1 (38.4–57.6)
*ω*(EC_50_), %	404 (109)	453 (4.0–1.3^6^)
*σ*, %	24.3 (5.8)	24.1 (20.9–27.1)
Platelet count		
BASE, 10^9^/L	242 (5.4)	243 (221–267)
MTT, h	173 (8.6)	172 (129–201)
*E*_max_	0.14 (7.1)	0.17 (0.08–0.37)
EC_50_, ng/mL	64.9 (33.3)	94.6 (34.4–269)
POW	0.19 (13.6)	0.19 (0.09–0.28)
GAM	Fixed to 1 (NA)	Fixed to 1 (NA)
*ω*(BASE), %	34.2 (20.7)	33.6 (27.0–41.0)
*ω*(EC_50_), %	167 (33.2)	122 (68.5–232)
*σ*, %	16.7 (2.9)	16.3 (14.4–18.7)
White blood cell count		
BASE, 10^9^/L	6.1 (6.0)	6.1 (5.3–6.9)
MTT, h	230 (6.3)	229 (167–272)
*E*_max_	0.1 (10.2)	0.1 (0.06–0.19)
EC_50_, ng/mL	7.1 (77.5)	7.1 (0.2–69.1)
POW	0.28 (16.9)	0.28 (0.13–0.43)
GAM	Fixed to 1 (NA)	Fixed to 1 (NA)
*ω*(BASE), %	42.8 (20.8)	42.5 (33.2–51.7)
*ω*(EC_50_), %	240 (62.6)	242 (45.2–558)
*σ*, %	26.0 (2.9)	25.7 (22.3–29.4)

*BASE* baseline endpoint value, *CI* confidence interval, *EC*_*50*_ concentration at half maximum effect, *GAM* Hill coefficient in the sigmoid, *E*_*max*_ effect model, *k*_*out*_ output/elimination rate constant, *k*_*PD*_ effect first-order rate constant, *MTT* mean transit time, *NA* not available, *POW* exponent on the feedback loop function, *RSE* relative standard error, *σ* residual variability, *ω* interindividual variability

**Fig. 2:**
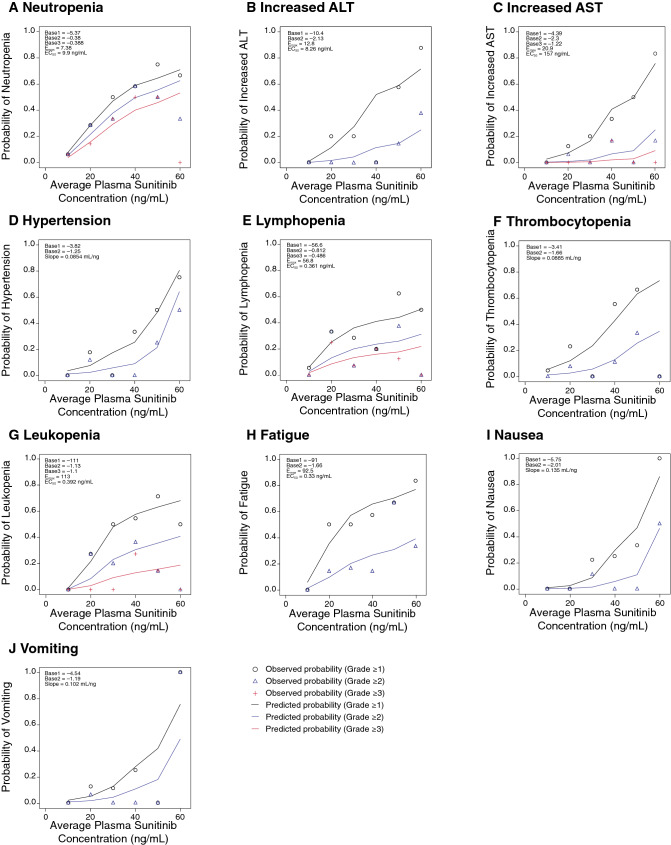
Observed and predicted probabilities of worst adverse event grades for the safety and tolerability endpoints. Probabilities of worst grade adverse events for **a** neutropenia (*n* = 23 patients), **b** increased ALT (*n* = 15), **c** increased AST (*n* = 13), **d** hypertension (*n* = 10), (**E**) lymphopenia (*n* = 16), **f** thrombocytopenia (*n* = 13), **g** leukopenia (*n* = 20), **h** fatigue (*n* = 24), **i** nausea (*n* = 7), and **j** vomiting (*n* = 7). *ALT* alanine transaminase, *AST* aspartate transaminase

Logistic regression analysis was used to determine the relationship between the incidences of the worst AE grade for each safety and tolerability endpoint and the calculated average plasma sunitinib concentration. The incidences of observed worst grade ≥ 1 AEs were neutropenia (*n* = 23 patients), increased ALT (*n* = 15), increased AST (*n* = 13), hypertension (*n* = 10), decreased hemoglobin (*n* = 1), lymphopenia (*n* = 16), thrombocytopenia (*n* = 13), and leukopenia (*n* = 20). When patients were stratified by average plasma sunitinib concentration (< median vs. ≥ median, respectively), incidences were neutropenia [17.2% (5/29 patients) vs. 60.0% (*n* = 18/30)], increased ALT [6.9% (2/29) vs. 43.3% (*n* = 13/30)], increased AST [3.4% (1/29) vs. 40.0% (*n* = 12/30)], hypertension [0% vs. 33.3% (*n* = 10/30)], lymphopenia [13.8% (4/29) vs. 40.0% (*n* = 12/30)], thrombocytopenia [3.4% (1/29) vs. 40.0% (*n* = 12/30)], and leukopenia [24.1% (7/29) vs. 56.7% (*n* = 17/30)]. There was a higher probability of neutropenia, increased ALT, increased AST, hypertension, lymphopenia, thrombocytopenia, and leukopenia with higher average plasma sunitinib concentrations (Fig. 2a–g). Because there was only one incident of decreased hemoglobin, logistic regression analysis could not be performed for this endpoint.

For the categorical safety and tolerability endpoints of fatigue, nausea, vomiting, intracranial hemorrhage, and hand-foot syndrome, PK–PD modeling was not used, but the association between plasma exposure and incidence was explored when there was a sufficient number of AEs. The incidences of observed worst grade ≥ 1 AEs were fatigue (*n* = 24 patients), nausea (*n* = 7), vomiting (*n* = 7), intracranial hemorrhage (*n* = 5), and hand-foot syndrome (*n* = 1). When patients were stratified by average plasma sunitinib concentration (< median vs ≥ median, respectively), the incidences were fatigue [24.1% (*n* = 7/29) vs. 56.7% (*n* = 17/30)], nausea [0% vs. 23.3% (*n* = 7/30)], vomiting [3.4% (*n* = 1/29) vs. 20.0% (*n* = 6/30)], intracranial hemorrhage [3.4% (1/29) vs. 13.3% (*n* = 4/30)], and hand-foot syndrome [0% vs. 3.3% (*n* = 1/30)]. Using logistic regression analysis, there was a higher probability of fatigue, nausea, and vomiting with higher average plasma sunitinib concentrations (Fig. 2h–j). Because there were only five incidents of intracranial hemorrhage and one incidence of hand-foot syndrome, the logistic regression analysis could not be performed for these endpoints.

## Discussion

In this population analysis of pooled data from pediatric patients with solid tumors administered sunitinib at 15–20 mg/m^2^ on schedule 4/2, the PK of sunitinib and SU012662 were well described using a two-compartment model with first-order absorption and *t*_lag_, using nonlinear mixed-effect modeling approaches. Covariate analysis identified BSA as the only statistically significant (*P* < 0.001) covariate for CL/F and Vc/F in the final models for both sunitinib and SU012662. Higher BSA was associated with greater CL/F and Vc/F, meaning lower sunitinib and SU012662 exposure. As part of the PK–PD analyses, the PK–PD models well described the time course of each safety and tolerability endpoint. Furthermore, no covariates were identified as statistically significant on either EC_50_ or k_PD_, indicating that the PK–PD relationship did not appear to be affected by body size or any other baseline characteristic, including age, sex, respective baseline safety endpoint values, or baseline Eastern Cooperative Oncology group performance status. Use of exposure–response logistic regression analysis showed that for each of the safety endpoints that had sufficient number of events to conduct and achieve a successful analysis, there was a higher probability of events with higher average sunitinib plasma concentrations. For hemoglobin decreased, hand-foot syndrome, and intracranial hemorrhage, there were too few events to conduct or achieve a successful logistic regression analysis.

To the best of our knowledge, this is the first population PK–PD analysis of sunitinib in a pediatric patient group. Therefore, these results cannot be directly compared with previous findings in other pediatric populations. However, several similar studies have been conducted in adult patient populations. In a study by Houk et al. [14] in adults with advanced solid tumors, including metastatic renal cell carcinoma (RCC) and gastrointestinal stromal tumor (GIST), there was a higher probability of fatigue and diastolic hypertension, and lower ANC, with greater sunitinib exposure (25–150 mg/day or every other day). In a second study by Lindauer et al. [15] in healthy adult volunteers, PK–PD models were successfully built for systolic and diastolic BP. In a third study by Khosravan et al. [16] in adult patients with advanced RCC or GIST, similar safety PK–PD relationships were identified as those shown in the current study in pediatric patients.

This study had some limitations. The dataset was relatively small, which sometimes led to large *η*-shrinkage values in EC_50_ and *k*_PD_ for some of the safety and tolerability endpoints. Therefore, failure to identify any statistically significant covariates on EC_50_ and *k*_PD_ should be interpreted with caution. In addition, the small sample size and the sampling schedule may have contributed to the wide range of bootstrap results for sunitinib Vp/F and SU012662 Q/F in the final PK models. In the PK–PD modeling and exposure–response analysis portions, there were too few events for some safety endpoints to explore fully the relationship between the incidence of AEs and sunitinib exposure. Finally, patients in the original clinical trials (studies ADVL0612 and ACNS1021) predominantly had high-grade glioma, ependymoma, brain stem glioma, or sarcoma [8–10]. Therefore, no patients in the current study had indications for which sunitinib is approved (e.g., RCC or GIST) albeit in adults [5, 6].

In conclusion, the PK of sunitinib and SU012662 was described using a two-compartment model with first-order absorption and *t*_lag_, and BSA was the only covariate that statistically significantly affected CL/F and Vc/F. Higher BSA was associated with lower sunitinib and SU012662 exposure. No covariates statistically significantly affected parameters in the PK–PD models. For each of the safety endpoints with a sufficient incidence of events to conduct or achieve a successful analysis/model, there was a higher probability of AEs with higher average plasma sunitinib concentrations. The exposure–response relationships of safety endpoints of sunitinib in pediatric patients with solid tumors were mainly driven by sunitinib plasma exposures and were not affected by age, sex, respective baseline safety endpoint values, baseline Eastern Cooperative Oncology Group performance status, or body size.

## Electronic supplementary material

Below is the link to the electronic supplementary material.

Supplementary file1 (EPS 492 kb)

Supplementary file2 (EPS 24322 kb)

Supplementary file3 (EPS 17930 kb)

Supplementary file4 (EPS 23559 kb)

Supplementary file5 (EPS 25943 kb)

Supplementary file6 (EPS 458 kb)

Supplementary file7 (DOCX 13 kb)

## Data Availability

Upon request, and subject to certain criteria, conditions and exceptions (see https://www.pfizer.com/science/clinical-trials/trial-data-and-results for more information), Pfizer will provide access to individual de-identified participant data from Pfizer-sponsored global interventional clinical studies conducted for medicines, vaccines and medical devices (1) for indications that have been approved in the US and/or EU or (2) in programs that have been terminated (i.e., development for all indications has been discontinued). Pfizer will also consider requests for the protocol, data dictionary, and statistical analysis plan. Data may be requested from Pfizer trials 24 months after study completion. The de-identified participant data will be made available to researchers whose proposals meet the research criteria and other conditions, and for which an exception does not apply, via a secure portal. To gain access, data requestors must enter into a data access agreement with Pfizer.
