# Supplementary material for: Population pharmacokinetics–pharmacodynamics of sunitinib in pediatric patients with solid tumors
Source: Cancer Chemother Pharmacol. 2020 Jul 4;86(2):181–92. doi: 10.1007/s00280-020-04106-z (PMC7417410; doi:10.1007/s00280-020-04106-z)
Supplement: Supplementary file 7 — Supplementary file7 (DOCX 13 kb) [file 280_2020_4106_MOESM7_ESM.docx]

**ONLINE RESOURCE**

**METHODS**

**Eligibility and exclusion criteria for study ADVL0612**

Study ADVL0612 consisted of two parts. In the first part, pediatric patients received sunitinib capsules [1]. However, young children may have a limited ability to swallow capsules, so in the second part patients received sunitinib as a powder formulation sprinkled on yogurt or applesauce [2]. Patients were eligible to participate in both parts if the following criteria were met: aged 2─21 years; histologic diagnosis of solid malignancy with measurable or evaluable disease, and no known curative options; Karnofsky (age >10 years) or Lansky (age ≤10 years) performance score ≥50; to have recovered from prior therapy; adequate baseline bone marrow, renal, hepatic, pancreatic, and cardiac function according to the protocol; stable thyroid function in patients with pre-existing hyper- or hypothyroidism; blood pressure <95^th^ percentile for age, height and sex; and not receiving concomitant antihypertensive medications.

Exclusion criteria for both parts of the study included the following: concurrent use of strong CYP3A4 inducers or inhibitors; treatment with agents that might increase the risk of bleeding complications; presence of pleural based tumors; or uncontrolled infection.

For the capsule part of the study only, patients were excluded if they could not swallow capsules and if they had body surface area <0.5m^2^. In addition, an amendment to the eligibility criteria was made after enrollment of the first 12 patients, because of cardiac and hematologic toxicity. For the remainder of the capsule study, patients with prior anthracycline treatment or cardiac radiation exposure were excluded. In addition, patients with known bone marrow metastases were excluded to provide a clear assessment of the hematological toxicity of sunitinib after 1 patient among the original 12 participants developed dose limiting neutropenia. The protocol amendment also permitted the enrollment of patients with central nervous system disorders who had been excluded initially.

For the powder formation part of the study only, additional exclusion criteria included: prior anthracycline or cardiac radiation exposure; allergy or intolerance to both yogurt and applesauce; and patients with known central nervous system primary tumors or metastatic disease if they demonstrated any evidence of tumor-associated hemorrhage.

**Eligibility and exclusion criteria for study ACNS1021**

Patients were eligible for study ACNS1021 [3] if they met the following criteria: age 18 months to 22 years; histological confirmation of high-grade glioma or ependymoma with evidence of measurable disease progression; performance status corresponding Eastern Cooperative Oncology Group scores of 0–2; two or fewer prior treatment regimens; intervals from prior therapy to enrollment of two weeks or more for focal (salvage) radiation therapy, 24 weeks for full field radiotherapy, and 3–6 weeks for myleosuppressive antitumor therapy, depending on specific agent and count recovery; no prior therapy with sunitinib or another targeted inhibitor of VEGF, PDGF, or KIT pathways; for patients receiving dexamethasone therapy, a stable or decreasing dose for at least 7 days prior to enrollment; ability to take oral medication; and adequate bone marrow and organ function.

Exclusion criteria included: use of enzyme-inducing antiepileptic medications within 7 days prior to enrollment; prior radiation therapy that included the mediastinal region; prior therapy with known risk for cardiovascular complications, eg anthracycline therapy; history of cerebrovascular accident, recent hemorrhage, or other significant thromboembolic event within 12 months prior to enrollment.

**References for Online Resource Methods**

1. Dubois SG, Shusterman S, Ingle AM, Ahern CH, Reid JM, Wu B, Baruchel S, Glade-Bender J, Ivy P, Grier HE et al (2011) Phase I and pharmacokinetic study of sunitinib in pediatric patients with refractory solid tumors: a children's oncology group study. Clin Cancer Res 17:5113–5122. doi: 10.1158/1078-0432.CCR-11-0237

2. DuBois SG, Shusterman S, Reid JM, Ingle AM, Ahern CH, Baruchel S, Glade-Bender J, Ivy P, Adamson PC, Blaney SM (2012) Tolerability and pharmacokinetic profile of a sunitinib powder formulation in pediatric patients with refractory solid tumors: a Children's Oncology Group study. Cancer Chemother Pharmacol 69:1021–1027. doi: 10.1007/s00280-011-1798-2

3. Wetmore C, Daryani VM, Billups CA, Boyett JM, Leary S, Tanos R, Goldsmith KC, Stewart CF, Blaney SM, Gajjar A (2016) Phase II evaluation of sunitinib in the treatment of recurrent or refractory high-grade glioma or ependymoma in children: a children's Oncology Group Study ACNS1021. Cancer Med 5:1416–1424. doi: 10.1002/cam4.713
